# Hair hormone profiling as a non-invasive diagnostic approach for assessing long-term endocrine status and productivity in Hereford bulls

**DOI:** 10.14202/vetworld.2025.3761-3778

**Published:** 2025-12-10

**Authors:** Alexey Frolov, Oleg Zavyalov, Zulfiya Galieva

**Affiliations:** 1 Department of Beef Cattle Breeding and Beef Production Technology, Federal Research Centre of Biological Systems and Agrotechnologies of the Russian Academy of Sciences, Orenburg, Russia; 2 Department of Technology of Meat, Dairy, Bashkir State Agrarian University, Ufa, Russia

**Keywords:** beef cattle, cortisol, endocrine biomarkers, hair hormones, Hereford bulls, meat quality, productivity, somatotropin

## Abstract

**Background and Aim:**

Accurate evaluation of hormonal status is critical for optimizing growth performance and meat quality in beef cattle. Conventional matrices such as blood, saliva, and urine reflect only short-term fluctuations and are influenced by collection stress. Hair, as a retrospective biosubstrate, can integrate hormone secretion over time and serve as a non-invasive indicator of chronic endocrine activity. This study aimed to evaluate the relationship between hair hormone concentrations and productive performance in Hereford bulls and to establish reference intervals (RIs) for major hormones in hair.

**Materials and Methods:**

A total of 200 Hereford bulls aged 15–18 months were reared under uniform feeding and housing conditions. Hair samples from the withers were processed into powder (d50 < 20 µm), and concentrations of 12 hormones, including cortisol, adrenaline, testosterone, estradiol, somatotropin (STH), thyroxine (T4), and insulin, were quantified by enzyme-linked immunosorbent assay. Slaughter data included live and carcass weights, yields, and physicochemical meat traits. Correlation analyses (Spearman) and stepwise multiple linear regression were used to determine hormonal predictors of productivity. RIs (2.5^th^–97.5^th^ percentiles) were calculated according to American Society for Veterinary Clinical Pathology recommendations.

**Results:**

Hair and serum hormones correlated significantly only for STH (r = 0.69) and adrenocorticotropic hormone (r = 0.61). Cortisol and adrenaline were inversely related to anabolic hormones (testosterone and insulin) and showed negative associations with live weight gain, carcass weight, and meat yield, but positive associations with meat pH and lipid oxidation. STH, insulin, estradiol, testosterone, and T4 were positively related to growth rate, carcass composition, and protein content. Regression models identified STH as the strongest independent positive predictor (β = 0.49) and cortisol as the principal negative predictor (β = –0.35) of productivity. RIs for 12 hormones were established for diagnostic application.

**Conclusion:**

Hair hormone analysis reliably reflects chronic endocrine status and predicts productive performance in beef cattle. Elevated stress hormones impair growth and meat quality, whereas anabolic hormones enhance carcass traits. The established RIs can serve as practical benchmarks for metabolic monitoring and herd management strategies in precision beef production.

## INTRODUCTION

Hormones are biologically active compounds secreted by endocrine glands and specialized cell groups in various tissues. They play a pivotal role in the humoral regulation of physiological processes such as metabolism, growth, reproduction, immune response, and maintenance of homeostasis [1, 2]. In beef cattle production, the endocrine system is a key regulatory axis that determines growth performance and overall productivity. Anabolic hormones, including somatotropin (STH), insulin-like growth factor-1 (IGF-1), and testosterone, stimulate protein synthesis and muscle accretion, while thyroid hormones (triiodothyronine [T3] and thyroxine [T4]) regulate basal metabolic rate and energy balance [3]. Conversely, cortisol, the principal stress hormone, mobilizes energy reserves and exerts catabolic effects; however, chronic elevation can suppress growth, reduce weight gain, and impair meat quality. Because hormonal imbalances directly influence productivity, health, and reproductive efficiency, precise evaluation of hormonal status has become an essential component of modern animal husbandry [4, 5].

Conventionally, biological matrices such as blood [6] and saliva [7] have been used to assess hormonal profiles in livestock, yet these substrates have notable limitations. Hormone concentrations exhibit pronounced short-term variability influenced by diurnal rhythm, feeding, and handling stress. Moreover, saliva sampling in cattle is technically demanding, whereas blood collection is invasive and itself induces stress responses that can distort results, particularly for cortisol and catecholamines. Urine analysis also presents practical challenges, as single collections provide limited diagnostic value due to circadian fluctuations, and long-term sampling is difficult under extensive housing systems [8].

Hair has emerged as an alternative biosubstrate that overcomes these drawbacks. It offers non-invasive and simple sampling, convenient storage and transport, and long-term stability of analytes without loss of diagnostic value [9]. Importantly, hair serves as a retrospective matrix, integrating hormonal secretion over weeks or months and thereby reflecting chronic endocrine activity rather than transient fluctuations. This makes it suitable for identifying long-term metabolic or stress-related disorders [10]. Although hair hormone analysis is increasingly used in human and biomedical research to monitor chronic stress and endocrine function, its application in animal production remains limited. Most studies in livestock have examined hair cortisol levels in dairy cows, focusing on associations with milk yield, somatic cell count, and housing conditions [11]. However, research on beef cattle is scarce and largely focused on a few hormones, predominantly cortisol, leaving a substantial knowledge gap regarding the broader hormonal profile and its relationship with meat productivity and quality [12].

Despite significant advances in endocrine physiology and analytical methodologies, the practical application of hair hormone analysis in beef cattle remains largely unexplored. Most available studies have focused on dairy cows, where hair cortisol has been correlated with milk yield, udder health, and housing systems. However, in beef production systems, research addressing the relationship between long-term hormonal status and economically important traits such as growth rate, carcass yield, and meat quality is extremely limited. The few existing reports are generally confined to cortisol measurement, overlooking the broader endocrine interactions among anabolic (STH, testosterone, insulin, and T4) and catabolic (cortisol and adrenaline) hormones that collectively determine productivity outcomes. Moreover, no comprehensive dataset currently exists describing the reference intervals (RIs) for multiple hormones in the hair of beef bulls, even though such baseline information is essential for implementing non-invasive hormonal diagnostics in herd management. This lack of standardized reference values and the absence of integrated hormone–productivity models have restricted the wider adoption of hair analysis as a reliable tool for endocrine and performance monitoring in beef cattle.

The present study was designed to fill these critical knowledge gaps by conducting a comprehensive assessment of hair hormone profiles in Hereford bulls during the fattening period. The specific objectives were as follows:

1. To quantify the concentrations of key anabolic, catabolic, thyroid, and reproductive hormones in the hair of Hereford bulls and to validate the reliability of this biosubstrate for endocrine assessment
2. To determine the relationships between hair hormone levels and major indicators of meat productivity and quality, including growth rate, carcass yield, composition, and physicochemical characteristics of meat
3. To establish RIs for 12 essential hormones in bovine hair according to the recommendations of the American Society for Veterinary Clinical Pathology (ASVCP).

By achieving these objectives, the study aims to develop a scientifically grounded, non-invasive diagnostic framework for evaluating chronic hormonal status in beef cattle. This approach will contribute to improving herd health, optimizing fattening efficiency, and enhancing meat quality through better understanding of the endocrine mechanisms that regulate productivity.

## MATERIALS AND METHODS

### Ethical approval

Animals were maintained and experimental studies were conducted in accordance with the instructions and recommendations of normative acts: *Model Law of the Interparliamentary Assembly of the Member States of the Commonwealth of Independent States “On the Treatment of Animals,”* Article 20 (Resolution of the MA of the CIS Member States No. 29-17 dated 31.10.2007). The Local Ethics Committee of Orenburg State University, Orenburg, Russia, approved the protocol for the present investigation (Protocol No. 832, May 17, 2024). The research was conducted to minimize animal suffering and to reduce the number of subjects tested.

### Study period and location

The study was conducted from October 2024 to January 2025 at the Agrosakmara Farm and the Orenbiv Meat Processing Plant in the Southern Urals, Russia.

### Animals used and study design

The subjects of the study were purebred Hereford bull calves aged 15–18 months (n = 200; average body weight at 15 months: 412.2 ± 17.6 kg), reared on a single farm in the Orenburg Region of the Southern Urals, Russia. Ear tags were applied to both ears for identification, with an individual number marked using a chemical marker. This study was conducted during the winter housing period (October–January). The bulls selected for observation had an average daily gain of at least 900 g/day over 12–15 months.

### Housing and its management

The bulls were housed in a loose-housing system in accordance with traditional beef cattle production technology. After weaning, the bulls were transferred to a feedlot, where they were kept without a leash until 18 months of age. The feedlots were equipped with unheated shelters for rest and protection from inclement weather. The indoor space allowance was 6–8 m^2^/head. The floors were covered with deep, non-replaceable bedding (straw), which was topped up as it became soiled. The temperature, lighting regime, and air humidity inside the shelter were not controlled. Animals were housed in groups of 50/pen.

The bulls were fed on exercise-feeding pads adjacent to the shelters, which the animals could freely access. Clay mounds covered with non-replaceable straw bedding were installed at the center of the feeding pad. The exercise area allowance was 25 m^2^/head. A group feeder (feeding front: 80 cm per head; barrier height: 60 cm) was installed on the feeding pad. Group waterers with heated water (one waterer per pen section) were placed on the feeding pads. The water was sourced from a well.

Throughout the entire fattening period, stress was minimized. Animal handling was performed by a permanent, trained staff. Animal transportation (including delivery to the feedlot and dispatch for slaughter) was performed only once during the bulls’ entire life cycle. The group composition remained stable after formation, during the fattening period to prevent social stress. The availability of sufficient feedlot space and an optimal feeding front eliminated competitive stress.

Vaccination and deworming protocols were performed in accordance with the farm’s standard schedules. The bulls were vaccinated against infectious bovine rhinotracheitis, bovine viral diarrhea, parainfluenza-3, bovine respiratory syncytial virus, pasteurellosis, and clostridial infections. Deworming (against nematodes and cestodes) was conducted before the fattening period began. Hair and blood sampling were performed no <3 weeks after any vaccination or anthelmintic treatment to exclude their influence on short-term hormonal status.

### Feeding regimen

The steers were fed a mono-mixed diet distributed 3 times a day. The mono-mixture consisted of 60% concentrates (crushed corn, barley, and premix) and 40% roughage/succulent feeds (Sudan grass hay and alfalfa haylage). These feeds contained the following (% of dry matter): Metabolizable energy – 10.3–10.5 MJ; crude protein – 12.0%–12.5%; crude fiber – 15.0%–16.0%; and nitrogen-free extractives – 48%–54%. The basal diet composition was identical for all study animals and developed according to the feeding standards established for bulls during the 15–18 months fattening period [13]. The animals received no additional hormonal preparations.

The daily ration contained: Calcium – 65.1–66.2 g; Potassium – 98.4–108.9 g; Magnesium – 24.5–27.3 g; Sodium – 29.2–29.7 g; Phosphorus – 45.7–48.2 g; Cobalt – 7.8–8.2 mg; Chromium – 5.7–6.2 mg; Copper – 96.3–112.6 mg; Iron – 1615–1750 mg; Iodine – 6.9–7.8 mg; Manganese – 620–662 mg; Selenium – 2.6–3.1 mg; Zinc – 401–451 mg; Boron – 93.4–102.3 mg; Silicon – 376.0–419.9 mg; Lithium – 5.1–5.6 mg; Nickel – 13.7–15.7 mg; Vanadium – 0.9–1.0 mg; Arsenic – 0.59–0.65 mg; Aluminum – 288.1–320.6 mg; Strontium – 248.5–270.2 mg; Lead – 7.52–8.21 mg; Tin – 0.54–0.58 mg; Cadmium – 0.52–0.57 mg; Mercury – 0.003–0.003 mg; Vitamins: A– 150,000–180,000 IU/day; D3 – 22,500–30,000 IU/day; and E– 290–315 mg/day.

### Ambient conditions

The ambient air temperature during the study period was as follows: November: average daytime –5°C to –10°C, nighttime –10°C to –15°C; December: average daytime –8°C to –12°C, nighttime –13°C to –18°C; and January: average daytime –10°C to –14°C, nighttime –15°C to –20°C. The indoor temperature was 6–10°C higher than the outdoor temperature.

### Hair collection and preparation

Hair samples were collected from the upper wither’s region (minimum 0.4 g) once before shipment to the abattoir [14]. Hair was clipped using a Heiniger Saphir (Switzerland) animal clipper with stainless steel blades (cut height: 1.5 mm). Blades were treated with 96% ethanol before each sampling to prevent external contamination. All sampling procedures were performed using single-use Elegreen VINYLTEP TPE (Elegreen, Malaysia) rubber gloves. Hair was clipped close to the skin, and samples were stored in dry paper envelopes in a dark place at room temperature (18°C–24°C) until preparation commenced.

Sample cleaning followed this protocol: soaking in distilled water at 40°C–60°C for 3 h; a 2-h treatment in a 40% ethanol solution under simultaneous ultrasound exposure (frequency: 35 kHz, power: 300–450 W, amplitude: 10 mm); and a 2-h treatment in bidistilled water with ultrasound (parameters identical to the previous step). After cleaning, the hair was pulverized using an IMC vMILL05 vibration mill (IMC, Russia) with a steel grinding assembly. The particle size of the resulting powder, expressed as the distribution median (d50), was 20 µm. Hormone extraction from the hair was performed according to a previously described method for humans and monkeys [15].

#### Steroid hormones

25 mg of pulverized hair was weighed into a microcentrifuge tube. 500 µL of methanol was added to each sample, and the tubes were incubated for 24 h at 37°C. The samples were then placed in an ultrasonic bath for 10 min. Subsequently, they were centrifuged for 60 s at 16,300 × *g* in a microcentrifuge (DLab Scientific, China). 300 µL of each methanol extract was aliquoted into a new tube and dried under a nitrogen stream at 38°C. Dried extracts were reconstituted with 200 µL of phosphate buffer.

#### Non-steroid hormones

Pulverized hair (25 mg) was weighed into a microcentrifuge tube. 500 µL of phosphate buffer (pH ~7.0-7.4) was added to each sample, and the tubes were incubated for 24 h at 37°C. The samples were then placed in an ultrasonic bath for 10 min. Subsequently, they were centrifuged for 60 s at 16,300 × *g* in a microcentrifuge. 200 µL of each extract was pipetted into a new tube. All samples were analyzed within 10 days of collection. Freezing was not permitted.

### Hormone determination

Hormone levels in hair and blood serum samples were determined using enzyme immunoassay with an automatic microplate analyzer (Infinite F200 PRO, Tecan, Austria). All analyses were performed in triplicate. Before using a new kit, reagent validation was performed by analyzing calibration curves (R^2^ ≥ 0.99, coefficient of variation [CV] of calibrators <15%) and comparing the optical density of control samples with the manufacturer’s ranges. Table 1 presents the enzyme-linked immunosorbent assay (ELISA) Kit (IDEXX Laboratories, Inc., USA) validation data for the measured hormones.

**Table 1:** Characteristics of enzyme-linked immunosorbent assay reagent kits for hormone determination in biological samples.

Hormone	Sensitivity	Measurement range	Reproducibility (CV)
Cortisol	<0.05 ng/mL	0.1–1000 ng/mL	CV <8% - CV <10%;
Testosterone	<0.05 ng/mL	0.1–110 ng/mL	CV <15%
Somatotropin	<0.01 ng/mL	0.01–100 ng/mL	CV <15%
Thyroid-stimulating hormone	<0.001 ng/mL	0.001–50 ng/mL	CV <10%–CV <12%
Adrenocorticotropic hormone	<1 pg/mL	5–1000 pg/mL	CV <10%–CV <12%
Follicle-stimulating hormone	<0.01 ng/mL	0.01–100 ng/mL	CV <15%
Progesterone	<0.1 ng/mL	0.1–50 ng/mL	CV <15%
Adrenaline	0.1 ng/mL	0.1–100 ng/mL	CV <10%–CV <12%
Insulin	<0.001 pg/mL	0.001–2500 pg/mL	CV <10%– CV <12%
Thyroxine	<20 ng/mL	20–300 ng/mL	CV <15%
Triiodothyronine	<0.5 ng/mL	0.5–8 ng/mL	CV <15%
Estradiol	<1 pg/mL	5–5000 pg/mL	CV <15%

CV = Coefficient of variation.

Commercial ELISA kits validated for cattle hair extracts are currently unavailable; therefore, we used kits validated by the manufacturer for cattle serum and other biological fluids. The following steps were taken to ensure the reliability of measurements in the new matrix: For each kit, we assessed parallelism (serial dilutions of hair extracts were parallel to the calibration curve), accuracy (92%–108% recovery), and precision (intra- and inter-assay CV <12%). The quantification limits for all hormones were below the physiological ranges detected in the study. The validation data confirmed the suitability of the kits for the reliable quantification of hormones in cattle hair.

### Blood sample collection

Blood samples (9 mL) were collected once in the morning from the tail vein into vacuum blood collection tubes containing a clotting activator (Hebei Xinle Sci & Tech Co., Ltd., Hebei, China). Three tubes were collected per animal. Double-ended medical needles (1.2 × 38 mm) were used for blood collection. Blood sampling was performed before loading the animals for transport to the abattoir. Animals were restrained by the neck in a hydraulic restrainer, ensuring secure, non-injurious fixation in a comfortable position and preventing sudden movements or falls. The procedure was performed by an experienced veterinarian familiar with bull anatomy to ensure quick and accurate blood collection. The presence of strangers and ambient noise was minimized. Blood samples were placed in racks and transported to the research site in a thermal container to avoid freezing and light exposure. Serum was separated by centrifuging samples for 10 min at 700 × *g* after 24 h of collection. Subsequently, the blood was placed in a refrigerator and stored at 4°C.

### Slaughter and carcass evaluation

The bulls were transported to the meat-processing plant on the day preceding slaughter. The transport distance was 80 km, and the total transportation duration, including loading and unloading, was 4–5 h. Shipping was performed using a cattle truck equipped with forced-air ventilation. The loading density was not less than 3 m² of truck floor space per head. The truck floor was covered with straw bedding. To minimize stress during loading, portable shields were used, and no electric prods were applied. Transportation was conducted during daylight hours at an ambient air temperature of −12°C to −15°C, preventing both heat and cold stress.

On arrival at the meat-processing plant, the animals were placed in the pre-slaughter holding area, where they remained for 24 h. The bulls were kept in the same stable groups as during the fattening period, with a stocking density of not less than 3 m^2^/head. They had free access to water, but feeding was discontinued 24 h before slaughter (pre-slaughter fasting) in accordance with the plant’s technological regulations. Conditions in the holding area were controlled: the air temperature was maintained between 8°C and 12°C, and the supply-and-exhaust ventilation system remained operational. The animal handling staff were trained in low-stress techniques, and both noise levels and unauthorized personnel were minimized.

The pre-slaughter live weight (PSW) was measured individually after the 24-h fasting period. The animals were stunned using a stiletto, which pierced the medulla oblongata in the atlanto-occipital region. After clinical death was confirmed, the carcasses were suspended and exsanguinated according to the standard method. Hot carcass weight (HCW) and internal fat weight (IFW) were recorded after slaughter. Carcass and slaughter yields were calculated using the following standard formulas:

Carcass yield (%) = (HCW/PSW) × 100

Where HCW = Hot carcass weight (kg), PSW = Pre-slaughter weight (kg).

Slaughter yield (%) = (HCW+IFW)/PSW × 100

Where HCW = Hot carcass weight, IFW = Internal fat weight, and PSW = Pre-slaughter weight.

Slaughter was performed on the day following the animals’ arrival at the plant. Stunning was achieved by inserting the stiletto between the occipital bone and the atlas vertebra, after which the carcasses were suspended on an overhead rail by the hind leg. Exsanguination was carried out by severing the carotid arteries and jugular veins in the neck region. All slaughtering and carcass-dressing operations were conducted by highly qualified personnel in strict compliance with sanitary, hygienic, and product-safety standards at every production stage.

Carcasses were chilled for 24 h at 4 °C and then reweighed to determine chilled carcass weight. The chilling process was continuously monitored.

Stage 1 (Intensive chilling): Air temperature = −10°C to −12°C; air velocity = 1–3 m/s; duration = 1.5–3 h; target = surface temperature drop to approximately 0°C.

Stage 2 (Tempering): Air temperature = 0°C to +2°C; relative humidity = 90%–95%; air velocity = 0.1–0.3 m/s; target = deep muscle temperature (*M. longissimus dorsi*, *M. semimembranosus*, depth 7–8 cm) reaching 4°C.

Calibrated needle thermistors (selectively, n = 5 carcasses per batch, 2-h interval) and automated chamber microclimate-monitoring systems (temperature and humidity, continuous) were used. The completion criterion for chilling was achieving a deep-muscle temperature of 4°C within ≤ 24 h.

### Meat quality analyses (pH, composition, amino acids, and fatty acids)

The pH of the *longissimus dorsi* muscle (LDM) was measured 24 h post-slaughter using a portable pH meter equipped with a penetrating electrode 52-00 (Crison Instruments, SA, Spain). The probe was inserted into a small incision in the right LDM between the 7th and 9th ribs. After every five samples, the pH meter was recalibrated with two standard buffer solutions (pH 7.02 and 4.00) [16].

After chilling, the meat (lean) was separated from bones, tendons, and ligaments. All carcass parts were weighed, and their yields were calculated. The lean-to-bone ratios were determined using the following formulas:

Flesh-to-bone ratio = FW/BW

Where FW = flesh weight (kg) and BW = bone weight (kg).

Flesh-to-bone and connective tissue ratio = FW/(BW + TLW)

Where FW = flesh weight (kg); BW = bone weight (kg); TLW = tendon and ligament weight (kg).

Meat from the left side of each carcass was minced using an industrial meat grinder. The resulting mince was thoroughly mixed, and five spot samples from different locations were combined into a single pooled sample weighing 400 g, which was analyzed in triplicate [17]. In addition, a 200-g sample of the LDM was collected from the same side before boning. This muscle sample was taken transversely in the loin region at the level of the 9th–11th ribs. Meat samples were vacuum-packed and delivered to the research site. All samples were analyzed within 3 days of collection to avoid freezing.

The quality characteristics of the *LDM* and pooled mince samples were evaluated according to standard methodological recommendations. Freezing of samples was not permitted.

Moisture content: determined by drying samples in an oven at 100°C (SPU ShS-80-01, Russia).

Fat content: determined by Soxhlet extraction using hexane [18].

Protein content: determined by the Kjeldahl method [19].

Ash content: determined by dry ashing, charring, and subsequent incineration in a muffle furnace at 550 ± 25°C until constant mass [20].

Water-holding capacity (WHC) was determined according to Antipova *et al*. [21]. Aliquots (5.00 ± 0.01 g) were evenly applied to the inner surface of a 10 mL centrifuge tube using a glass rod. Tubes were hermetically sealed, inverted, and incubated in boiling water for 15 min. The expelled moisture mass was read from the tube’s graduation marks. WHC, expressed as a percentage, was calculated as:

WHC = M − MRA

Where WHC = water-holding capacity; M = mass fraction of moisture in the sample (%); MRA = moisture-releasing ability of meat (%).

The moisture mass fraction (M) in the samples was determined according to State Standard R 51479-99 [22]. Samples were dehydrated to a stable weight at (103°C ± 2°C), and the weight difference before and after drying was recorded.

Moisture-releasing ability was calculated as:

MRA = (a/m) × 100

Where a = amount of moisture released (mL); m = weight of sample (g).

Energy value of the flesh was calculated using the equation of V. M. Alexandros [23]:

Energy value (MJ per kg flesh) = ([P × 4.1] + [F × 9.3]) × 0.04187

Where P = protein (%); F = fat (%); 4.1 and 9.3 are energy-conversion coefficients for protein and fat, respectively; and 0.04187 is the kcal-to-MJ conversion factor.

Amino acid composition was analyzed in triplicate. The LDM was examined for oxyproline and tryptophan contents. Hydrolysis: acid hydrolysis (110°C, 14–16 h) for oxyproline; alkaline hydrolysis (110°C, 14–16 h) for tryptophan. Acid hydrolysates were filtered using blue-ribbon filters (Melior XXI LLC, Russia); alkaline hydrolysates were not filtered. All hydrolysates were derivatized with sodium carbonate (JSC Bashkir Soda Company, Russia) and phenylisothiocyanate (Shandong Hailan Chemical Industry Co., Ltd., China), dried under warm air (60–70°C), dissolved in 0.5 mL distilled water, and centrifuged at 2,800 × *g* for 5 min.

Capillary-electrophoresis analysis of the supernatant was performed using the *Kapel* system (Lumex-Marketing LLC, Russia) [24, 25]. System performance was controlled before each analytical series and periodically during measurements using a standard amino acid mixture at known concentrations containing target analytes. Performance-acceptance criteria included: stability of key amino acid migration times (variation ≤0.5–1.0%), resolution of critical peak pairs (threonine/serine, R ≥1.5), capillary efficiency >100,000 theoretical plates/m, peak symmetry (Asymmetry factor As ≤1.5), detector-response linearity in the working range (R^2^ ≥0.995), and reproducibility of internal-standard peak areas (norleucine or α-aminobutyric acid; relative standard deviation [RSD] ≤5 %).

Control analyses of the amino acid mixture were performed at least once per 10 samples (or at start/middle/end of series) to verify quantification accuracy, assess system precision, and calibrate the instrument.

Fatty acid composition of meat was determined in triplicate by gas chromatography using a Crystal-4000 Lux chromatograph (Lumex-Marketing LLC, Russia). Lipids were extracted using the Folch method (chloroform–methanol, 2:1). Transesterification was achieved by treating lipids with BF3–methanol (14 %, 100°C, 45 min).

Chromatographic performance and analytical stability were monitored daily before and during each analytical series using a certified standard mixture of fatty acid methyl esters (FAME mix) covering the expected analyte retention range. Performance-acceptance criteria included achievement of required resolution for critical peak pairs, conformity of retention times (± 0.05 min from set values), peak symmetry (Asymmetry factor < 1.5), detector-response linearity within the working range (R^2^ > 0.995), and reproducibility of internal-standard peak area (C17:0) (RSD < 5 %). Regular analysis of the FAME mix (at least once per 10 samples) ensured method accuracy, precision, and proper calibration.

All analyses were conducted at the laboratory of the Federal Research Center for Biological Systems and Agricultural Technologies of the Russian Academy of Sciences, Orenburg, Russia (certificate of accreditation № RA.RU.21ПФ59 dated 02.12.2024 г.).

### Statistical analysis

Testing the data distribution with the Shapiro–Wilk test indicated a deviation from normality. Consequently, the non-parametric Spearman’s rank correlation coefficient was applied for the correlation analysis, as it is robust to normality violations and the presence of outliers in the data. p < 0.05 was considered statistically significant. The RIs for chemical element concentrations (2.5^th^ and 97.5^th^ percentiles) in hair were calculated according to the recommendations of the ASVCP [26]. During sample analysis, values within the ranges <Q1 – 1.5× interquartile range (IQR) and > Q3 + 1.5× IQR were considered outliers and manually removed. The 90% confidence interval (CI) was calculated using the robust method described by Horn *et al*. [27]. In addition, the 25th and 75th percentiles were calculated according to the recommendations of Miroshnikov *et al*. [28]. The sample size was determined based on a previous study by Lovakov and Agadullina [29], which demonstrated reliable results with comparable effect sizes when assessing small to medium correlation strengths. Stepwise multiple linear regressions were applied to identify independent predictors of key productive traits. The model included the concentrations of all 12 hormones measured in hair as independent variables. The most significant indicators identified in the correlation analysis were the dependent variables: Live weight at 18 months, average daily gain, carcass weight, meat yield, protein content in meat, and pH at 24 h. p < 0.05 served as the criterion for a variable’s inclusion in the model, and p > 0.10 was the criterion for its removal. Data processing was performed using the Statistica 20.0 software package (“StatSoft Inc.,” USA) and IBM Statistical Package for the Social Sciences version 28 (IBM Corporation, NY, USA) software.

## RESULTS

### General characteristics of the animals

Analysis of the data obtained during the observation period showed that the bulls were physiologically healthy, had a stable hormonal profile, and exhibited relatively high average daily gains. This positively influenced their slaughter characteristics and the quality of the meat obtained. A more detailed description of the animals is presented in Table 2.

**Table 2:** Hormonal status, productivity, and meat quality of experimental Hereford bull calves.

Indicator	Meaning
Hormone content in the hair of Hereford bull calves aged 18 months	Cortisol – 28.71 ± 4.31 ng/g; adrenaline – 1.89 ± 0.12 ng/g; testosterone – 18.44 ± 2.35 ng/g; STH – 0.542 ± 0.11 ng/g; thyroglobulin – 0.091 ± 0.01 ng/g; ACTH – 14.95 ± 1.04 pg/g; FSH – 0.052 ± 0.01 ng/g; progesterone – 18.29 ± 2.88 ng/g; insulin – 0.016 ± 0.009 pg/g; T_4_ – 182.34 ± 7.68 ng/g; TSH – 2.80 ± 0.05 ng/g; estradiol – 19.64 ± 1.08 pg/g
Blood serum hormone levels of Hereford bull calves aged 18 months	Cortisol – 57.89 ± 8.36 ng/mL; adrenaline – 5.78 ± 0.29 ng/mL; testosterone – 15.83 ± 2.28; STH– 17.86 ± 4.26 ng/mL; thyroglobulin – 1.94 ± 0.34 ng/mL; ACTH – 135.93 ± 8.79 pg/mL; FSH – 1.20 ± 0.07 ng/mL; progesterone – 16.64 ± 2.21 ng/mL; insulin – 0.22 ± 0.09 pg/mL;T_4_ – 182.63 ± 6.9 ng/mL 1; TSH – 3.32 ± 0.13 ng/mL; estradiol – 68.36 ± 4.02 pg/mL
Live weight and average daily gains of Hereford bull calves	live weight at 15 months – 412.2 ± 17.6 kg; live weight at 18 months – 504.4 ± 21.5 kg; average daily gain (15-18 months) – 1013 ± 17.52 g/day
Slaughter control data for Hereford bulls	PSW – 492.97 ± 5.82 kg; HCW 283.5 ± 3.37 kg; carcass yield – 57.49 ± 0.392 %; IFW – 13.91 ± 0.247 kg; internal fat yield – 2.83 ± 0.154 %; HCW+IFW – 297.4 ± 3.04 kg; slaughter yield – 60.32 ± 0.343 %
Morphological characteristics of Hereford bull carcasses	Cold carcass weight – 279.6 ± 3.34 kg; FW – 223.2 ± 2.58 kg; flesh yield – 79.85 ± 0.291 %; BW – 46.69 ± 0.362 kg; bone yield – 16.71 ± 0.208 %; TLW – 9.62 ± 0.167 kg; tendon and ligament yield – 3.44 ± 0.225 %; FW/BW – 4.78 ± 0.154; FW/ (BW+TLW) – 3.96 ± 0.151
Nutritional properties of an average Hereford bull meat sample	Dry weight – 30.72 ± 0.595 %; protein – 18.84 ± 0.326 %; fat – 10.85 ± 0.144 %; ash – 1.02 ± 0.071 %; protein per carcass– 42.07 ± 1.31 kg; fat per carcass – 24.22 ± 0.581 kg; energy value of 1 kg of flesh – 7.46 ± 0.155 MJ; energy value of the entire carcass flesh – 1665.2 ± 15.54 MJ
Nutritional properties of the LDM muscle of Hereford bulls	Dry weight – 23.88 ± 0.216 a%; protein – 21.23 ± 0.182 a%; fat – 1.65 ± 0.097 c%; pH – 5.67 ± 0.103; water-holding capacity of meat – 55.83 ± 0.545 %
Amino acid content in the LDM of Hereford bulls, % d	Tryptophan, mi/% 382.3 ± 8.06; oxyproline, m/% 54.85 ± 0.527; protein quality indicator 6.97 ± 0.111
Fatty acid composition (% of total fatty acids) in the LDM of Hereford bulls	Palmitic (C16:0) 24.46 ± 0.215; stearic (C18:0) 19.47 ± 0.291; myristic (C14:0) 2.28 ± 0.079; myristoleic (C14:1) 3.03 ± 0.192; palmitoleic (C16:1) 2.79 ± 0.072; oleic (C18:1) 41.85 ± 0.422; linoleic (C18:2) 4.14 ± 0.061; linolenic (C18:3) 0.565 ± 0.042; arachidonic (C20:4) 1.42 ± 0.039

ACTH = Adrenocorticotropic hormone, STH = Somatotropin, FSH = Follicle-stimulating hormone, TSH = Thyroid-stimulating hormone, TLW = Tendon and ligament weight, PSW = Pre-slaughter weight, FW = Flesh weight, BW = Bone weight, HCW = Hot carcass weight, IFW = Internal fat weight, T_4_ = Thyroxine, LDM: Longissimus dorsi muscle.

### Correlation between hair and blood hormones

A correlation analysis was performed between hormone concentrations in hair and serum to assess the informativeness of hair as a biosubstrate for evaluating hormonal status (Table 3). A significant positive correlation was observed between the biosubstrates for only two of the 12 hormones studied: STH (r = 0.69, p < 0.05) and adrenocorticotropic hormone (r = 0.61, p < 0.05).

**Table 3:** Correlation coefficients of hormone content in hair with hormone content in the blood of Hereford bull calves (n = 200).

Hormones	Cortisol (hair)	Adrenaline (hair)	Testosterone (hair)	STH (hair)	TSH (hair)	ACTH (hair)	FSH (hair)	Progesterone (hair)	Insulin (hair)	T4 (hair)	T3 (hair)	Estradiol (hair)
Cortisol (blood)	−0.68*	0.52*	0.00	−0.02	−0.31*	0.04	−0.07	0.34*	0.08	−0.20	0.12	0.14
Adrenaline (blood)	0.67*	−0.55*	−0.01	−0.04	−0.32*	0.03	−0.07	0.35*	0.09	−0.20	0.12	0.14
Testosterone (blood)	−0.49*	−0.46*	0.34	−0.04	0.40*	0.03	−0.01	−0.48*	−0.08	0.10	−0.23	0.01
STH (blood)	0.03	−0.05	0.04	0.69*	0.09	0.05	−0.02	−0.03	0.23	−0.06	0.03	0.14
TSH (blood)	−0.35*	0.23	0.15	0.03	0.24	0.01	0.01	0.42*	0.10	0.06	0.15	0.02
ACTH (blood)	0.46*	0.62*	0.11	−0.14	−0.24	0.61	0.13	0.51*	0.04	0.04	0.19	−0.30*
FSH (blood)	0.00	−0.11	0.19	0.09	−0.04	0.12	0.17	0.12	0.27*	0.05	−0.02	−0.02
Progesterone (blood)	−0.06	−0.07	0.06	0.08	0.11	0.06	−0.11	−0.04	−0.31*	−0.01	−0.35*	0.08
Insulin (blood)	0.13	−0.01	−0.17	0.00	−0.04	0.07	0.13	0.12	0.31	0.00	−0.06	−0.09
T4 (blood)	0.07	−0.10	0.22	0.26*	−0.17	0.16	0.01	0.20	0.15	0.01	0.05	0.07
T3 (blood)	−0.45*	−0.46*	−0.07	0.09	0.37*	0.36*	−0.07	−0.50*	0.03	0.02	−0.16	0.17
Estradiol (blood)	0.03	−0.06	0.18	0.05	−0.07	0.14	0.09	0.14	0.39*	0.06	0.01	−0.04

* Reliable correlation. ACTH = Adrenocorticotropic hormone, STH = Somatotropin, TSH = Thyroid-stimulating hormone, FSH = Follicle-stimulating hormone, T_4_ = Thyroxine, T_3_ = Triiodothyronine.

Cortisol and adrenaline levels were consistently negatively correlated with their blood concentrations (r = −0.55 to −0.68, p < 0.05). For the remaining hormones, no significant linear dependence was found, or the correlations were weak and statistically insignificant.

These data indicate that hormone concentrations in hair only partially reflect their serum levels and primarily characterize long-term, cumulative hormonal status, in contrast to the short-term fluctuations recorded in blood.

### Interrelationships among hormones

Analysis of the relationships between the concentrations of 12 hormones in hair revealed statistically significant correlations, reflecting long-term interactions in the endocrine system of the bull calves (Table 4).

**Table 4:** Correlation coefficients of hormone content in the hair of Hereford bull calves (n = 200).

Hormones	Cortisol (hair)	Adrenaline (hair)	Testosterone (hair)	STH (hair)	TSH (hair)	ACTH (hair)	FSH (hair)	Progesterone (hair)	Insulin (hair)	T4 (hair)	T3 (hair)	Estradiol (hair)
Cortisol (hair)	1.00	0.81*	0.31*	−0.15	−0.31*	0.27*	0.11	0.23	−0.44*	0.07	0.17	−0.34*
Adrenaline (hair)	0.81*	1.00	0.19	−0.24	−0.26*	0.34*	0.13	0.67*	−0.03	0.02	0.18	−0.22
Testosterone (hair)	0.31*	0.19	1.00	0.05	−0.17	0.07	−0.05	0.46*	0.24	−0.03	0.23	0.13
STH (hair)	−0.15	−0.24	0.05	1.00	0.17	0.10	0.07	−0.06	0.41*	−0.10	0.16	0.32*
TSH (hair)	−0.31*	−0.26*	−0.17	0.17	1.00	0.12	0.14	−0.43*	−0.17	−0.03	−0.21	−0.12
ACTH (hair)	0.27*	0.34*	0.07	0.10	0.12	1.00	−0.09	−0.20	−0.19	−0.02	−0.31*	0.38*
FSH (hair)	0.11	0.13	−0.05	0.07	0.14	−0.09	1.00	0.08	0.05	−0.08	0.16	−0.62*
Progesterone (hair)	0.23	0.67*	0.46*	−0.06	−0.43*	−0.20	0.08	1.00	0.15	0.01	0.24	−0.16
Insulin (hair)	−0.44*	−0.03	0.24	0.41*	−0.17	−0.19	0.05	0.15	1.00	−0.15	0.41*	0.17
T4 (hair)	0.07	0.02	−0.03	−0.10	−0.03	−0.02	−0.08	0.01	−0.15	1.00	0.20	−0.08
T3 (hair)	0.17	0.18	0.23	0.16	−0.21	−0.31*	0.16	0.24	0.41*	0.20	1.00	−0.07
Estradiol (hair)	−0.34*	−0.22	0.13	0.32*	−0.12	0.38*	−0.62*	−0.16	0.17	−0.08	−0.07	1.00

* Reliable correlation. TSH = Thyroid-stimulating hormone; STH = Somatotropin; ACTH = Adrenocorticotropic hormone; FSH = Follicle-stimulating hormone; T4 = Thyroxine; T3 = Triiodothyronine.

The most pronounced positive correlation was observed between the stress-response markers cortisol and adrenaline (r = 0.81, p < 0.05). Cortisol also exhibited significant positive associations with adrenocorticotropic hormone (ACTH) (r = 0.27, p < 0.05) and progesterone (r = 0.23, p < 0.05), but negative associations with testosterone (r = −0.31, p < 0.05), insulin (r = −0.44, p < 0.05), and estradiol (r = −0.34, p < 0.05).

In addition to its strong association with cortisol, adrenaline correlated positively with ACTH (r = 0.34, p < 0.05) and showed the strongest positive correlation among all hormones with progesterone (r = 0.67, p < 0.05).

STH (growth hormone [GH]) demonstrated positive relationships with insulin (r = 0.41, p < 0.05) and estradiol (r = 0.32, p < 0.05), indicating a potential synergistic anabolic effect of this hormone group. Among the reproductive hormones, the expected positive correlation was recorded between testosterone and progesterone (r = 0.46, p < 0.05). Thyroid-stimulating hormone (TSH) correlated negatively with cortisol (r = −0.31, p < 0.05), adrenaline (r = −0.26, p < 0.05), and progesterone (r = −0.43, p < 0.05). The strongest negative correlation in the dataset was found between follicle-stimulating hormone (FSH) and estradiol (r = −0.62, p < 0.05).

Overall, the resulting pattern of interactions confirms that the hormonal profile in hair reflects complex and long-term endocrine interconnections, where stress hormones (cortisol and adrenaline) act antagonistically toward key anabolic and reproductive hormones such as testosterone, insulin, and STH.

### Impact of hair hormone levels on productivity and meat quality

Correlation analysis revealed a complex set of significant relationships between hormone concentrations in bulls’ hair and their performance traits (Tables 5-8).

**Table 5:** Correlation coefficients of hair hormone content with productive and slaughter indicators of bull calves (n = 200).

Indicators	A	B	C	D	E	F	G	H	I	J	K	L
Live weight at 15 months (kg)	−0.73*	−0.78*	0.31	0.33	0.02	−0.47*	0.52*	0.37*	0.1	0.15	0.13	0.58*
Live weight at 18 months (kg)	−0.69*	−0.43*	0.53*	0.64*	0.41*	−0.64*	0.26	0.14	0.29	0.17	−0.07	0.55*
Absolute gain (kg)	−0.71*	−0.07	0.52*	0.66*	0.48*	−0.56*	0.25	−0.21	0.38*	0.29	−0.15	0.61*
Average daily gain (15–18 months) (g)	−0.71*	−0.07	0.52*	0.66*	0.48*	−0.56*	0.25	−0.21	0.38*	0.29	−0.15	0.61*
Pre-slaughter weight (kg)	−0.59*	−0.63*	0.31	0.14	0.21	−0.14	0.26	0.14	0.39*	0.17	−0.07	0.61*
Hot carcass weight (kg)	−0.56*	−0.50*	0.3	0.15	0.22	−0.09	0.25	0.12	0.37*	0.19	−0.08	0.59*
Internal fat weight (kg)	0.51*	0.2	−0.30*	−0.45*	0.22	−0.09	0.25	0.12	0.43*	0.19	−0.08	0.51*
Cold carcass weight (kg)	−0.52*	−0.43*	0.3	0.45	0.23	−0.10	0.25	0.12	0.29	0.19	−0.07	0.42*
pH	0.61*	0.1	−0.25	0.19	−0.31	0.25	−0.04	−0.18	0.09	−0.17	−0.27	−0.20
Thiobarbituric number	0.52*	0.48*	0.33	−0.42*	−0.23	−0.18	0.02	0.12	0.32	−0.37*	−0.04	−0.13
Flesh weight (kg)	−0.48*	0.31	0.41*	0.52*	0.14	0.24	0.11	0.12	0.45*	0.18	−0.08	0.39*
Flesh yield (%)	−0.41*	0.25	0.39*	0.55*	0.18	0.21	0.14	0.14	0.44*	0.2	−0.09	0.41*
Bone weight (kg)	−0.28	0.17	0.17	0.39*	0.23	−0.22	−0.09	0.18	0.19	0.19	−0.09	0.33
Bone yield (%)	−0.32	0.21	0.25	0.37*	0.17	−0.18	−0.11	0.17	0.1	0.21	−0.08	0.29
Tendon & ligament weight (kg)	−0.31	−0.12	0.18	0.44*	0.08	0.05	0.07	0.12	0.31	0.18	0.04	0.09
Tendon & ligament yield (%)	−0.28	−0.09	0.22	0.37	0.06	0.03	0.02	0.11	0.24	0.11	0.04	0.11
Slaughter yield (%)	−0.15	−0.12	0.18	0.37	0.07	0.09	0.05	0.08	0.18	0.22	0.07	0.17

* Reliable correlation, A = Cortisol (hair), B = Adrenaline (hair), C = Testosterone (hair), D = Somatotropin (hair), E = Thyroid-stimulating hormone (hair), F = Adrenocorticotropic hormone (hair), G = Follicle-stimulating hormone (hair), H = Progesterone (hair), I = Insulin (hair), J = Thyroxine (hair), K = Triiodothyronine (hair), L = Estradiol (hair).

**Table 6:** Correlation coefficients of hormone content in hair with indicators of the chemical composition of minced meat of 18-month-old Hereford bull calves aged 18 months (n = 200).

Indicators (%)	A	B	C	D	E	F	G	H	I	J	K	L
Moisture	0.31*	0.49	−0.07	−0.66*	0.03	−0.31	−0.05	−0.24	0.16	0.08	−0.30	0.22
Dry weight	−0.31*	−0.49	0.07	0.66*	−0.03	0.31	0.05	0.24	0.16	−0.08	0.3	−0.22
Fat	0.03	−0.34	−0.35*	−0.46*	−0.25	0.24	−0.14	0.39	0.39*	−0.05	0.35	0.33*
Ash	0.07	0.36	−0.23	−0.26	0.29	−0.50	0.13	−0.36	0.02	0.07	−0.38	−0.02
Protein	−0.28	−0.31	0.19	0.43*	0.15	0.17	0.22	0.14	0.32	0.05	0.14	0.19
Energy value (1 kg flesh)	0.18	0.28	0.43	0.33	0.18	0.14	0.29	0.11	0.36*	0.22	0.34	0.17

* Reliable correlation. A = Cortisol (hair), B = Adrenaline (hair), C = Testosterone (hair), D = Somatotropin (hair), E = Thyroid-stimulating hormone (hair), F = Adrenocorticotropic hormone (hair), G = Follicle-stimulating hormone (hair), H = Progesterone (hair), I = Insulin (hair), J = Thyroxine (hair), K = Triiodothyronine (hair), L = Estradiol (hair).

**Table 7:** Correlation coefficients of hormone content in hair with fatty acid content in the longissimus dorsi muscle of 18-month-old Hereford bull calves.

Indicators (%)	A	B	C	D	E	F	G	H	I	J	K	L
Myristic acid	−0.20	−0.01	−0.26	−0.12	0.74*	0.2	0.13	−0.14	−0.48	0.18	−0.33	−0.46
Myristoleic acid	−0.07	−0.03	−0.20	0.05	−0.37	−0.58*	0.13	−0.16	0.14	−0.19	0.22	−0.14
Palmitic acid	0.07	−0.30	−0.27	0.13	0.24	0.04	−0.03	0.06	−0.31	−0.10	−0.51	0.18
Palmitoleic acid	−0.48	−0.30	−0.08	0.15	0.3	0	0.68*	−0.21	0.03	−0.65*	0.05	−0.61*
Stearic acid	0.01	0.26	−0.03	0.14	−0.19	−0.12	0.01	0.03	−0.24	0.56*	0.19	−0.10
Oleic acid	0.13	0.15	0.25	−0.42	−0.18	0.09	−0.13	−0.05	0.5	−0.23	0.18	0.12
Linoleic acid	−0.60*	0.14	−0.10	0.09	−0.19	−0.25	0.33	−0.16	−0.13	0.01	0.2	−0.22
Linolenic acid	−0.53*	0.55*	0.07	−0.04	−0.32	−0.61*	0.01	0.02	0.2	0.13	0.25	0.09
Arachidonic acid	0.14	0.08	−0.09	0.12	0.28	−0.44	−0.19	0.26	0.08	−0.22	−0.20	0.22

* Reliable correlation, A = Cortisol (hair), B = Adrenaline (hair), C = Testosterone (hair), D = Somatotropin (hair), E = Thyroid-stimulating hormone (hair), F = Adrenocorticotropic hormone (hair), G = Follicle-stimulating hormone (hair), H = Progesterone (hair), I = Insulin (hair), J = Thyroxine (hair), K = Triiodothyronine (hair), L = Estradiol (hair).

**Table 8:** Coefficients of correlation of hormone content in hair with indicators of the longissimus dorsi muscle chemical composition of Hereford bull calves at 18 months of age.

Indicators	A	B	C	D	E	F	G	H	I	J	K	L
Moisture (%)	−0.21	0.32	−0.13	−0.10	−0.33	−0.33	0.18	−0.28	−0.33	0.29	0.29	−0.20
Dry weight (%)	0.21	−0.32	0.13	0.30	0.33	0.33	−0.18	0.28	0.33	−0.29	−0.29	0.20
Fat (%)	0.24	−0.23	0.03	0.35	0.58*	0.46	−0.15	0.31	0.36*	−0.16	−0.27	0.39*
Protein (%)	−0.31	−0.28	0.17	0.36*	0.16	0.15	0.18	0.16	0.25	0.08	0.17	0.11
Ash (%)	−0.29	0.14	−0.02	−0.05	−0.63*	−0.42	0.13	−0.26	−0.09	0.04	0.23	−0.08
Glycogen (Unit)	−0.72*	−0.65*	0.28	0.29	0.53*	−0.32	0.25	0.14	0.00	−0.16	0.42	−0.02
Water-holding capacity of meat	0.11	0.15	0.08	0.21	0.09	0.18	0.22	0.32	0.14	0.08	−0.04	0.11

* Reliable correlation, A = Cortisol (hair), B = Adrenaline (hair), C = Testosterone (hair), D = Somatotropin (hair), E = Thyroid-stimulating hormone (hair), F = Adrenocorticotropic hormone (hair), G = Follicle-stimulating hormone (hair), H = Progesterone (hair), I = Insulin (hair), J = Thyroxine (hair), K = Triiodothyronine (hair), L = Estradiol (hair), WHC = Water holding capacity.

Stress hormones, cortisol and adrenaline, were found to be consistent negative predictors of productivity, showing significant negative correlations with live weight at 15 and 18 months (up to −0.73 and −0.69 for cortisol; −0.78 and −0.43 for adrenaline), absolute and average daily gain (up to −0.71), pre-slaughter weight, and carcass weight.

At the same time, elevated cortisol levels were associated with increased internal fat deposition (r = 0.51), higher meat pH (r = 0.61), and reduced muscle glycogen content (r = −0.72), collectively indicating a negative impact of cortisol on both productivity and meat quality.

In contrast, the anabolic hormones STH and testosterone showed positive associations with growth performance (live weight at 18 months: r = 0.64 and r = 0.53; weight gains: r = 0.66 and r = 0.52) and with muscle tissue development, as confirmed by the correlation of STH with meat yield (r = 0.52) and protein content in meat (r = 0.43 in mince and r = 0.36 in the LDM).

Interestingly, estradiol showed strong positive correlations with weight metrics (r = 0.55–0.61) and fat content (r = 0.33–0.39), suggesting a substantial role in male bovine metabolism, a phenomenon warranting further investigation.

Analysis of the fatty acid profile revealed specific, statistically significant associations: TSH was positively correlated with myristic acid (r = 0.74), while cortisol and adrenaline were negatively correlated with linoleic and linolenic acids.

Thus, measuring hormones in hair proves to be a valuable, non-invasive approach, allowing assessment of long-term hormonal status and predicting its influence on both animal productivity and the qualitative characteristics of meat.

### Identification of predictors of productive qualities

Stepwise multiple linear regression was conducted to identify the independent contribution of hair hormone concentrations to variation in key productive traits.

The model for live weight at 18 months, which included STH, cortisol, and estradiol, explained 61% of its variability (adjusted R^2^ = 0.61) (Table 9). The standardized regression coefficients (β) indicated that STH was the strongest positive predictor (β = 0.49), while cortisol exerted a significant negative effect (β = −0.35). Estradiol also made an independent positive contribution (β = 0.22).

**Table 9:** Multiple regression analysis of live weight and weight gain predictors in Hereford bulls.

Dependent variable	Independent predictor	Regression coefficient (β)	Standard Error	p-value	Standardized β
Live weight at 18 months (kg)	Constant	312.45	28.91	<0.001	–
	STH	24.81	5.23	<0.001	0.49
	Cortisol	−12.56	3.45	0.002	−0.35
	Estradiol	18.92	7.84	0.025	0.22
R² adjusted = 0.61					
Average daily increase (g/day)	Constant	845.33	89.12	<0.001	–
	STH	95.67	18.45	<0.001	0.52
	Cortisol	−58.34	14.21	0.001	−0.33
	Adrenaline	−42.11	19.87	0.047	−0.18
R² adjusted = 0.61					

STH = Somatotropin.

A similar pattern was observed for the average daily gain, where STH and cortisol remained the main predictors with opposing effects, while adrenaline emerged as an additional independent negative factor.

Carcass weight was most reliably predicted by STH (positive) and cortisol (negative) concentrations, with the model explaining 53% of the variance (Table 10). In addition to STH, meat yield was positively associated with insulin concentration, confirming its anabolic role in muscle tissue development.

**Table 10:** Multiple regression analysis of predictors of meat productivity and carcass yield in Hereford bulls.

Dependent variable	Independent predictor	Regression coefficient (β)	Standard error	p-value	Standardized β
Carcass weight (kg)	Constant	158.90	22.34	<0.001	–
	STH	+15.89	3.12	<0.001	0.51
	Cortisol	−9.87	2.58	0.001	−0.31
R² adjusted = 0.53					
Pulp yield (kg)	Constant	112.65	18.91	<0.001	–
	STH	+11.24	2.45	<0.001	0.48
	Insulin	+5.78	2.89	0.042	0.19
R² adjusted = 0.45					
Internal fat mass (kg)	Constant	8.45	3.21	0.015	–
	Cortisol	+2.11	0.78	0.014	0.28
	Estradiol	+1.98	0.85	0.029	0.25
R² adjusted = 0.32					

STH = Somatotropin.

Internal fat deposition was independently and positively associated with cortisol and estradiol levels, consistent with their known roles in lipogenesis and energy redistribution. Cortisol concentration was the main independent factor associated with increased meat pH at 24 h (a negative quality indicator), whereas STH exerted the opposite effect (Table 11).

**Table 11:** Multiple regression analysis of meat quality predictors of Hereford bull calves.

Dependent variable	Independent predictor	Regression coefficient (β)	Standard error	p-value	Standardized β
pH after 24 h	Constant	5.41	0.18	<0.001	–
	Cortisol	+0.15	0.04	0.002	0.40
	STH	−0.09	0.04	0.038	−0.22
R² adjusted = 0.38					
Protein content of meat (%)	Constant	18.12	1.45	<0.001	–
	STH	+1.25	0.31	0.001	0.41
	Testosterone	+0.89	0.38	0.028	0.21
R² adjusted = 0.41					
Fat content in meat (%)	Constant	12.34	2.11	<0.001	–
	Estradiol	+1.56	0.52	0.008	0.32
	Cortisol	+1.02	0.48	0.044	0.20
R² adjusted = 0.29					

STH = Somatotropin.

Protein content in meat was positively and independently correlated with STH and testosterone concentrations, confirming their role in stimulating muscle-protein synthesis. Intramuscular fat content was positively dependent on estradiol and cortisol levels.

Overall, the regression analysis identified STH as the most consistent independent positive predictor of meat productivity and quality, whereas cortisol served as the primary independent negative predictor. The effects of other hormones, such as estradiol, insulin, and testosterone, were more specific, influencing certain productivity or quality parameters.

### Establishment of RIs for hormone concentrations in hair

Considering the informativeness of hair for assessing hormonal status and its significant impact on the productive qualities of bulls, RIs for hormone concentrations in the hair of the experimental animals were calculated (Table 12). The obtained intervals are recommended for evaluating long-term hormone metabolism disorders in bulls during the fattening period.

**Table 12:** Reference intervals of the physiological norm of hormone concentrations in the hair of Hereford bull calves calculated in accordance with the recommendations of the American Society for Veterinary Clinical Pathology.

Hormones	Percentile	

	2.5 (90% Confidence interval)	97.5% (90% Confidence interval)
Cortisol, ng/g	0.55 (0.51–0.59)	50.54 (46.29–55.81)
Adrenaline, ng/g	0.28 (0.19–0.37)	3.97 (3.78–4.37)
Testosterone, ng/g	3.81 (3.72–3.90)	28.31 (26.08–33.53)
Somatotropin, ng/g	0.05 (0.03–0.08)	0.98 (0.87–1.21)
Thyroid-stimulating hormone, ng/g	0.05 (0.04–0.06)	0.13 (0.10–0.17)
Adrenocorticotropic hormone (pg/g)	1.71 (1.60–1.82)	30.22 (28.10–31.34)
Follicle-stimulating hormone, ng/g	0.02 (0.01–0.03)	0.07 (0.06–0.08)
Progesterone, ng/g	0.32 (0.25–0.39)	33.34 (31.06–35.62)
Insulin, pg/g	1.97 (1.59–2.35)	20.82 (19.91–21.23)
Thyroxine, ng/g	140.03 (128.56–151.50)	218.00 (210.43–229.57)
Triiodothyronine, ng/g	2.07 (1.88–2.20)	3.31 (3.18–3.54)
Estradiol, pg/g	10.08 (9.53–10.62)	27.51 (25.98–29.15)

The percentile ranking method used for RI calculation is based on ordering the entire range of observed values (100% of the sample) by magnitude. The ordered series is then divided into 100 equal percentile intervals, and RI boundaries are set at specific percentiles.

In this study, following the recommendations of the ASVCP, the physiological norm within the sample (after excluding outliers – abnormally high and/or low values) is proposed as the interval from the 2.5^th^ to the 97.5^th^ percentile. This method requires the calculation of 90% CIs for the upper (97.5%) and lower (2.5%) boundaries of the RI. These CIs estimate the range within which the true population boundary value would lie if the sample size were increased in the future, with 90% probability.

However, based on accumulated experience in medical and veterinary practice, RIs reflecting the central range of the physiological norm can also be calculated as the interval between the 25^th^ and 75^th^ percentiles of a representative sample.

Based on the results of this study, the following 25^th^ and 75^th^ percentile values for hormone concentrations in bull hair were established:

- Cortisol: 2.88–10.90 ng/g
- Adrenaline: 0.42–0.82 ng/g
- Testosterone: 7.17–12.30 ng/g
- STH: 0.24–0.42 ng/g
- TSH: 0.06–0.09 ng/g
- ACTH: 7.58–14.83 pg/kg
- FSH: 0.03–0.50 ng/g
- Progesterone: 0.20–15.66 ng/g
- Insulin: 2.76–4.74 pg/g
- T4: 176.77–191.14 ng/g
- T3: 2.29–2.58 ng/g
- Estradiol: 10.89–19.07 pg/g

These intervals provide practical reference values for assessing long-term endocrine function and identifying deviations in hormonal balance among beef bulls during the fattening phase.

## DISCUSSION

### Hair as a biosubstrate for assessing long-term hormonal changes

Hair is an informative biosubstrate for assessing long-term changes in the hormonal status of animals and humans [30]. Hormones are incorporated into hair primarily through the blood vessels surrounding the hair follicle during the growth phase: circulating hormones in the bloodstream passively diffuse from the capillaries into the matrix cells, and as keratinization and hair-shaft formation occur, the hormones become “sealed” within the structure [31]. Other probable, though less significant, routes of incorporation are thought to involve diffusion from sebaceous glands for lipophilic hormones and from sweat contacting the hair surface [32].

The development of a non-invasive method for long-term monitoring of hormonal status directly aligns with the “One Health” concept and the improvement of animal welfare. Hair-sample collection causes minimal disturbance compared with repeated blood sampling, thereby allowing more reliable physiological data collection, reducing the overall stress level, and facilitating more informed management decisions [33].

### Applicability of hair-based hormone measurement in cattle research

The methodology for measuring hormones in hair has previously been successfully applied by Ghassemi Nejad *et al*. [34] on dairy cattle, where it proved valuable for assessing chronic stress and its connection with health status and reproductive function. Our study confirmed that this method is highly informative for evaluating chronic hormonal status in beef bulls, with results consistent with previous data on the Hereford breed. Specifically, high hair cortisol concentrations were associated with reduced weight gain, lower meat productivity, and impaired meat quality [35].

The principal novelty of the present research lies in establishing, for the first time, extensive RIs for cortisol, testosterone, estradiol, GH, and other hormone concentrations in the hair of beef bulls. These findings provide a robust foundation for future research on endocrine regulation and its relationship to productivity in beef cattle.

### Experimental design and sample representativeness

Purebred Hereford bull calves were selected based on a weight gain intensity of at least 900 g/day during the preceding 3-month fattening period. This selection criterion was intentionally established because it aligns with the targets of intensive beef cattle production in the study region and serves as an indicator of good health and the full realization of genetic potential.

Such an approach enabled the formation of a productivity-homogeneous sample that accurately represented a commercial herd while minimizing the influence of hidden pathologies and metabolic disorders on the analysis of hormonal status. The uniformity of physiological condition in the test group thereby strengthened the reliability of the correlation and regression results.

### Biological significance of hormone–productivity relationships

The most valuable findings of this research are the established correlations between levels of chronic hormones and productive traits. Cortisol demonstrated consistent negative correlations with live weight gain and carcass weight, confirming its role as a marker of chronic stress, as well as a positive association with internal fat mass, which may be attributed to its influence on lipid metabolism and insulin resistance [36]. Furthermore, elevated cortisol levels were associated with increased meat pH at 24 h due to glycogen depletion in muscle tissue [37], leading to deterioration of meat quality and shelf life, and a reduction in essential fatty-acid content [38].

STH (GH) was positively correlated with meat yield, reflecting its role in stimulating protein synthesis through IGF-1 [39], while concurrently promoting lipolysis [40]. This dual function explains its inverse relationship with fat deposition.

Estradiol exhibited positive correlations with live weight indicators and carcass fat content, likely mediated by activation of the somatotropic axis [41] and direct receptor-mediated effects on muscle and adipose tissues [42]. Insulin and testosterone were also positively associated with growth performance and meat productivity, as insulin enhances glucose utilization and protein synthesis, whereas testosterone directly stimulates muscle-protein synthesis and myofiber hypertrophy [43].

### Hormonal interactions and fatty acid metabolism

When assessing interactions among hormones, a negative correlation was identified between cortisol and testosterone concentrations. This relationship can be explained by cortisol-induced suppression of the hypothalamic–pituitary–gonadal axis, competition for common steroidogenic enzymes, and a direct inhibitory effect on Leydig-cell function. A similar competitive mechanism involving the enzyme 11β-hydroxysteroid dehydrogenase type 1 may underlie the cortisol-mediated suppression of estradiol bioavailability in target tissues [44].

The relationships identified between hormones and the fatty acid profile are directly important to meat quality. The negative correlation between cortisol level and the proportion of polyunsaturated fatty acids (linoleic and linolenic acids) suggests enhanced β-oxidation under chronic stress, leading to reduced nutritional value of the meat. Conversely, positive associations between anabolic hormones and monounsaturated fatty acids (e.g., oleic acid) are favorable indicators of improved oxidative stability, extended shelf life, and enhanced sensory quality of the product [45].

## CONCLUSION

This study demonstrated that hair is a highly informative, non-invasive biosubstrate for assessing the long-term hormonal status of beef bulls. Analysis of 12 key hormones revealed that hair hormone concentrations reliably reflect chronic endocrine activity, complementing the short-term hormonal fluctuations typically captured in serum. Significant interrelationships among the hormones confirmed the existence of integrated endocrine regulatory networks, where stress-related hormones such as cortisol and adrenaline act antagonistically to anabolic and reproductive hormones, including STH, insulin, and testosterone.

Correlation and regression analyses provided strong evidence that STH serves as the primary independent positive predictor of growth rate, carcass weight, meat yield, and protein content, whereas cortisol functions as the principal negative predictor, being closely linked to reduced productivity, increased fat deposition, elevated meat pH, and diminished meat quality. Estradiol, insulin, and testosterone exhibited hormone-specific, mostly anabolic influences on productivity and composition traits. Furthermore, the negative associations of cortisol with essential polyunsaturated fatty acids and the positive relationships between anabolic hormones and monounsaturated fatty acids highlight the long-term impact of endocrine balance on meat nutritional and sensory properties.

From a practical perspective, these findings have immediate implications for precision livestock management. Hair-based hormonal profiling offers a cost-effective, animal-friendly alternative to repeated blood sampling, allowing farmers to identify stress-prone or low-performing individuals early and to optimize nutrition, housing, and handling conditions. The establishment of RIs for 12 hormones in beef-bull hair provides a scientifically grounded framework for physiological monitoring, welfare assessment, and selective breeding based on endocrine resilience and growth efficiency.

A major strength of the study lies in the large, physiologically homogeneous sample of Hereford bulls maintained under commercial fattening conditions, which ensured high internal validity of the results. However, limitations include the single-breed focus and the absence of longitudinal sampling across multiple production cycles, which may restrict broader generalization. Future research should expand the analysis to different breeds, production systems, and climatic zones, integrate molecular biomarkers of stress and metabolism, and investigate seasonal and nutritional modulation of hair hormone deposition dynamics.

The present study confirms that the endocrine profile of hair reflects the chronic hormonal background that underlies beef cattle productivity and meat quality. Incorporating this approach into herd management will enhance both animal welfare and production efficiency, aligning with the principles of the “One Health” framework and sustainable livestock farming.

## AUTHORS’ CONTRIBUTIONS

OZ: Mathematical processing of experimental data and drafted and revised the manuscript. FA: Methodology and supervised the study. GZ: Conducted the study and data analysis and interpretation. All authors have read and approved the final version of the manuscript.
